# Automatic Detection of Faults in Race Walking: A Comparative Analysis of Machine-Learning Algorithms Fed with Inertial Sensor Data

**DOI:** 10.3390/s19061461

**Published:** 2019-03-25

**Authors:** Juri Taborri, Eduardo Palermo, Stefano Rossi

**Affiliations:** 1 Department of Economics, Engineering, Society and Business Organization (DEIM), University of Tuscia, 01100 Viterbo, Italy; stefano.rossi@unitus.it; 2 Department of Mechanical and Aerospace Engineering, Sapienza University of Rome, 00184 Rome, Italy; eduardo.palermo@uniroma1.it

**Keywords:** race walking, illegal steps, machine-learning algorithms, inertial sensors, activity recognition

## Abstract

The validity of results in race walking is often questioned due to subjective decisions in the detection of faults. This study aims to compare machine-learning algorithms fed with data gathered from inertial sensors placed on lower-limb segments to define the best-performing classifiers for the automatic detection of illegal steps. Eight race walkers were enrolled and linear accelerations and angular velocities related to pelvis, thighs, shanks, and feet were acquired by seven inertial sensors. The experimental protocol consisted of two repetitions of three laps of 250 m, one performed with regular race walking, one with loss-of-contact faults, and one with knee-bent faults. The performance of 108 classifiers was evaluated in terms of accuracy, recall, precision, F1-score, and goodness index. Generally, linear accelerations revealed themselves as more characteristic with respect to the angular velocities. Among classifiers, those based on the support vector machine (SVM) were the most accurate. In particular, the quadratic SVM fed with shank linear accelerations was the best-performing classifier, with an F1-score and a goodness index equal to 0.89 and 0.11, respectively. The results open the possibility of using a wearable device for automatic detection of faults in race walking competition.

## 1. Introduction

Race walking is a long-distance discipline within the sport of athletics. It became a formal sporting event at the Olympics in 1960. During race walking, two technical requirements must be observed by the athletes. First, the athlete shall keep at least one foot on the ground, avoiding the flight phase, which is typical of running. The corresponding violation is called loss of contact (LC). The second possible violation is the knee-bent (KB), when the supporting leg, the one in contact with the ground, is not kept straight during the foot contact period [1]. An athlete is disqualified from the competition if he/she receives three warnings, regardless of the type of fault [2]. Several referees have to supervise the right race walking technique of each athlete during the competition. As of today, the judgment is based on the unaided human eye, thus lacking in objectivity and fueling debates during official events [3]. 

Two main flaws can be found in visible observations from race judges. Firstly, the human eye can retain an image rating at 16 Hz [4]. Thus, a flight phase lasting less than 0.06 s cannot be detected by any judge. In addition, athletes race in group, making the tracking of a single athlete extremely difficult, which is also known as change blindness theory [5]. Secondly, the judges are stationed at different points on the race circuit and therefore they can assess each athlete during restricted periods of time. The direct consequence of these flaws is the presence of missed or incorrect disqualifications during each official competition. Consequently, the credibility of this Olympic sport is decreasing more and more, and its inclusion among Olympic Games is still questioned [6]. From this perspective, the introduction of technological systems able to automatically detect illegal steps seems appealing for improving the accuracy of race walking judgments [7].

## 2. Related Works

In the last decades, activity recognition has gained great popularity among research groups [8,9,10,11]. Machine-learning algorithms have demonstrated their capability of optimal classifications in several fields, such as clinics [12,13], daily life activities [14,15], robotics [16], and sports applications [17,18]. Focusing on sports, Ermes et al. [17] applied a hybrid classifier, combining a decision tree and an artificial neural network (ANN), fed with data from accelerometers placed on the hip and wrist for discriminating among running, cycling, rowing, and playing football. In addition, Long et al. presented a Bayesian classifier for recognizing sports activities such as soccer, volleyball, badminton, table tennis, etc. [19]. Finally, Heinz et al. proposed an ANN for the detection of motion sequences in martial arts, with the aim of distinguishing among levels of experience and quality in executing movements [20]. 

Building on these findings, different studies have already been proposed for the automatic detection of illegal steps during race walking. Santoso et al. [21] proposed the use of two piezoelectric transducers placed on the sole of each shoe. The on/off status of the piezoelectric transducer allowed real-time detection of loss-of-contact faults. Lee et al. [22] assessed that only one linear accelerometer placed on the S1 vertebra yielded a sensitivity equal to 88% in detecting LC faults by estimating the flight time derived from the analysis of the vertical acceleration curve [23]. Di Gironimo and co-workers [24,25,26] validated the use of a single inertial sensor placed on the L5/S1 intervertebral space for the automatic detection of LC faults by means of a binary classifier, reaching a sensitivity equal to 82%. 

However, all of the abovementioned studies evaluated the feasibility of automatically identifying only LC faults without considering KB faults. Conversely, Ciu et al. [27] developed a device composed of two pressure sensors placed on the sole of each shoe to detect LC faults, along with two strain transducers embedded in two kneepads to monitor KB faults. However, it was evident that the kneepads could affect the normal gesture of athletes, thus losing its applicability during official competition. 

Hence, to the best of the authors’ knowledge, no studies have evaluated the feasibility of a machine-learning algorithm fed with data gathered from inertial sensors that can automatically discriminate both faults. This study aimed to validate and compare different machine-learning algorithms, fed with features related to linear acceleration and angular velocity of the lower-limb segments, for the automatic discrimination of both LC and KB faults. Towards this aim, we investigated both the most suitable machine-learning algorithm and the best body segment to place the sensor, as well as the most appropriate dataset of features with which to feed the algorithm. The validation of the proposed methodology might represent a starting point for the development of a wearable, noninvasive device to assist referees in race walking judgments, as well as to help race walkers to correct their technique during training.

## 3. Materials and Methods

### 3.1. Theoretical Background 

Machine-learning algorithms have increasingly gained more popularity in the last decades in the field of physical activity recognition [28]. Machine learning indicates the process of learning mathematical rules from a dataset of features related to different classes to create a classifier able to discriminate those classes when fed with a new dataset of features [29]. In this work, we focused on the supervised machine-learning classification: according to this approach, the classifier knows the classes of features during the training stage. Consequently, the choice of features that are significantly different among classes represents a crucial step in the development of any machine-learning classifier, and it is task-dependent [9]. 

Supervised classifiers can be divided into four main categories: geometric, binary, probabilistic, and template matching [9]. For the aim of this work, the first two appeared to be the most widespread due to their higher ability in discriminating physical human activity with respect to others [30]. A geometric classifier discriminates the classes by constructing decision boundaries for dividing the space of the features. The decision boundaries depend on geometric rules that are optimized during the training stage [31]. The binary classification process, instead, is articulated in several different steps to reach a binary decision following threshold-based detectors [9]. In this study, we tested the support vector machine (SVM), the k-nearest neighbor (kNN), and the artificial neural network (ANN) as geometric classifiers and the decision tree (DT) as a binary classifier. The choice was based on a review paper [30], which highlighted them as the most widespread in literature for physical human activity recognition.

No dynamic models, such as recurrent neural networks and long short-term memory, were tested, as the classification process during race walking only depends on the *i-th* stride, with no information brought by the previous one. Furthermore, the implementation of dynamic models is expected to impinge real-time classification, as it requires greater computational resources [32]. 

*Support Vector Machine*

**Figure 1:**
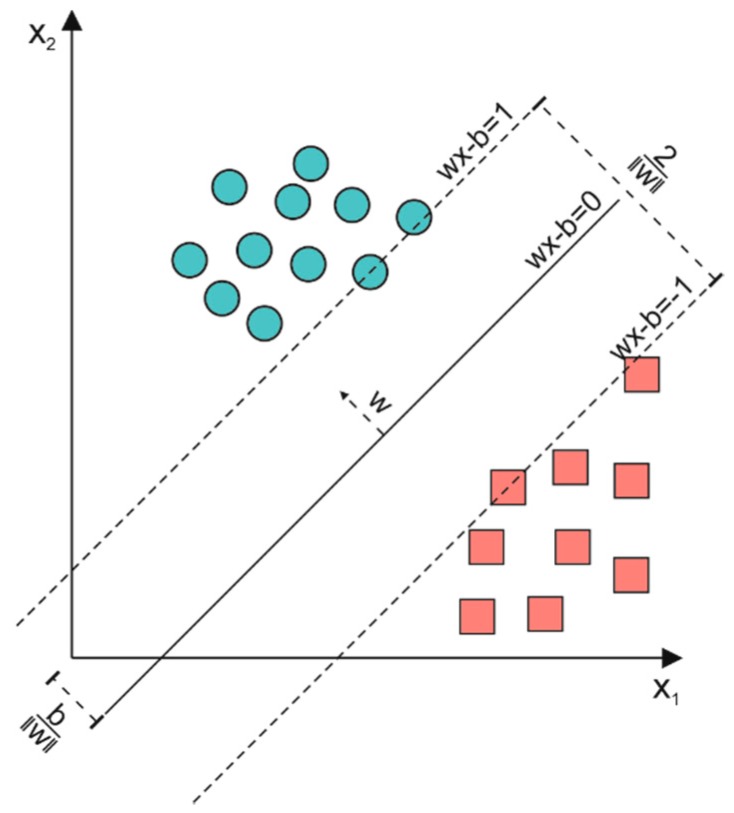
Example of a support vector machine (SVM). Circles and squares represent features related to two different classes. The distance 2/‖w‖ is the defined margin.

An SVM is a supervised geometric machine-learning algorithm and it is one of the most widespread in the classification process [33]. In this algorithm, each feature represents a point in an n-dimensional space (where n is the number of considered features), and the classification process is based on the identification of the hyperplane that separates the features related to different classes by maximizing the distance (w) between the hyperplane and the nearest points of the different classes. The main element of the SVM algorithm is the kernel function, which transforms a nonlinear feature space into a linear one before the search of the hyperplane. Based on the equation of the kernel function, several SVMs can be implemented; in particular, we tested three kernel functions: linear (SVM_l_), quadratic (SVM_q_), and cubic (SVM_c_). Regardless of the kernel function, all of the tested SVMs were implemented in this work with the same setting parameters, as follows: (i) box constraint level set to 1 in order to maximize the accuracy; (ii) multiclass method set to “one-vs.-one”; and (iii) auto kernel scale mode disactivated. Figure 1 shows an example of classification performed with SVM.

*k-Nearest Neighbor*

**Figure 2:**
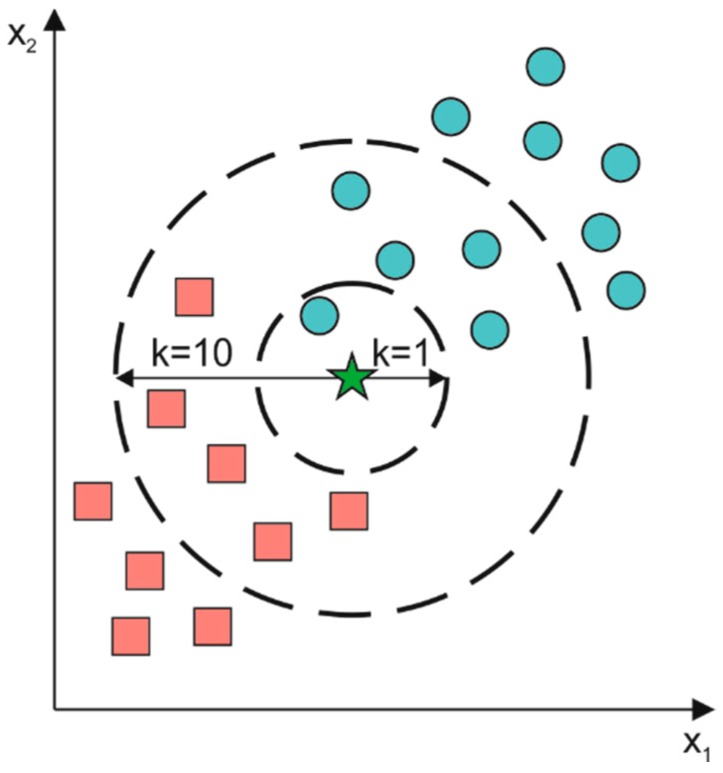
Example of a k-nearest neighbor (kNN) with the number of neighbors set to 1 or 10. k represents the computed distance. Circles and squares represent features related to two different classes. Star represents new data to classify.

A kNN algorithm is one of the simplest classification algorithms for activity recognition [34]. Each combination of measured features represents a point in an n-dimensional space, and the classification process is performed by identifying the most common class among the k-nearest neighbors by maximizing the distance among neighbors related to different classes. Based on the type of computed distance, several kNNs can be implemented; in particular, we tested: (i) a fine kNN (kNN_f_), which used the Euclidian distance to make distinctions between classes with the number of neighbors (k) set to 1 and an equal distance weight among the classes; (ii) a cosine kNN (kNN_c_), which used the cosine distance metric, with the number of neighbors set to 10 and an equal distance weight among the classes; (iii) a cubic kNN (kNN_cu_), which used the cubic distance metric (Minkowski metric), with the number of neighbors set to 10 and an equal distance weight among the classes; and, (iv) a weighted kNN (kNN_w_), which used a weighting Euclidian distance based on the squared inverse approach with the number of neighbors set to 10. An example of a kNN is shown in Figure 2. 

The distances were computed with the following equations:

(1) $$d=\sqrt{\sum_{i=1}^{k}{\left(x_{i}-y_{i}\right)}^{2}}$$

(2) $$d=\frac{x\cdot y}{∥x∥∥y∥}$$

(3) d=∑i=1k((|xi−yi|)q)1q

(4) $$d=\sqrt{\sum_{i=1}^{k}w_{i}{\left(x_{i}-y_{i}\right)}^{2}.}$$

In particular, Equations (1)–(4) were used to compute the Euclidean distance, the cosine distance, the cubic distance, and the weighted distance, respectively. 

*Artificial Neural Network*

**Figure 3:**
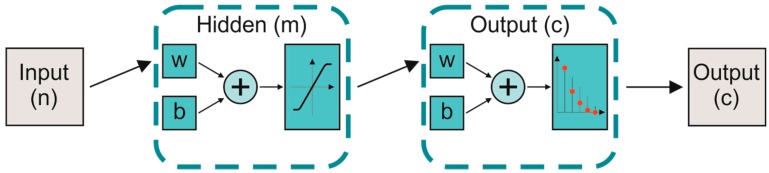
Scheme of an artificial neural network (ANN) classification. n, m, and c represent the number of input, hidden, and output layers, respectively. w and b represent the weights and biases related to the interconnections among neurons.

An ANN is a supervised geometric machine-learning algorithm inspired by y the information process within the nervous system [35]. The structure of an ANN is organized in layers, and it is composed of a large number of processing elements, called artificial neurons, interconnected with specific weights (w) and biases (b). Each artificial neuron is characterized by several weighted inputs, a transfer function, and one output. The number of the input neurons (n) is equal to the number of considered features, while the number of output layers (c) corresponds to the number of classes to discriminate. Hidden layers (m) are interposed between input and output neurons. In particular, we tested an ANN with three hidden layers, which represented a good trade-off between speed of classification and high level of accuracy [36]. In addition, the scaled conjugate gradient was used during the training stage and the convergence was evaluated up to 1000 iterations through entropy indices. The scheme of an ANN is shown in Figure 3. 

*Decision Tree*

**Figure 4:**
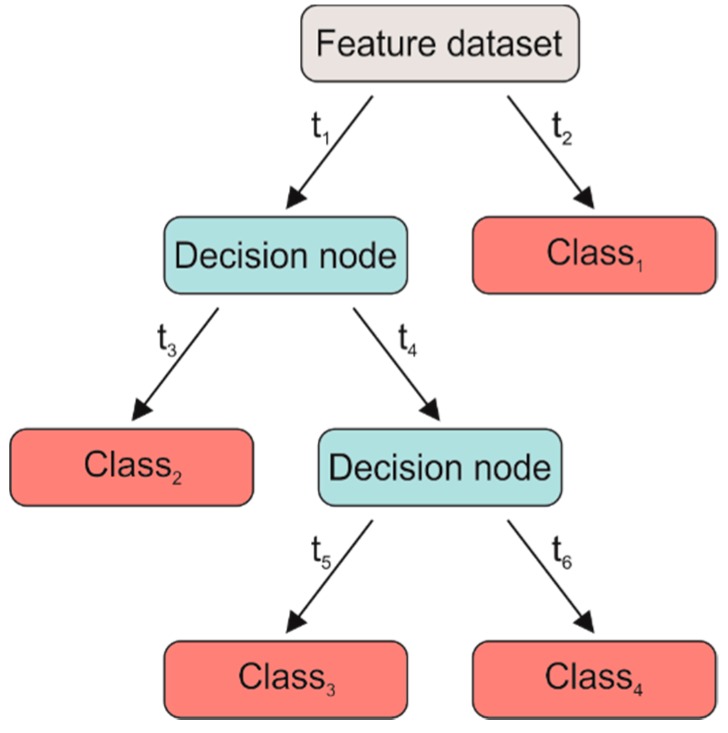
Scheme of a generic decision tree (DT). t_i_ represent the thresholds of the splitting. Grey rectangle represents the input, red ones represent the outputs, and blue ones represent the decision nodes where the rules of the classification are applied.

The DT is the main example of binary classifiers [37]. Binary classification consists of creating a DT in which each node discriminates between two classes, following different strategies based on threshold detectors (t). In particular, we tested the fine (or complex) DT (DT_f_), in which the maximum number of splits was set to 100. The split criterion was based on the Gini diversity index and surrogate decision splits were not allowed. An example of a DT is shown in Figure 4. 

### 3.2. Experimental Protocol

Eight expert race walkers (seven men, one woman, 21.0 ± 7.5 years) were enrolled. Written informed consent was obtained from the participants. The experimental procedure was performed at the Istituto di Medicina e Scienza dello Sport of the Italian National Olympic Committee. Athletes were enrolled if they had at least 5 years of experience in race walking and they were at least 14 years old to ensure the complete development of the mobility of the lower-limb joints [38]. All participants were not subject to injuries in the previous 2 years and they had not undergone any orthopedic surgeries.

Each subject was sensorized with seven inertial measurement units (IMUs, MTw, Xsens Technologies, Enschede, The Netherlands), embedding a triaxial linear accelerometer and a triaxial gyroscope. Some specifics related to the used IMUs are reported in Table 1.

**Figure 5:**
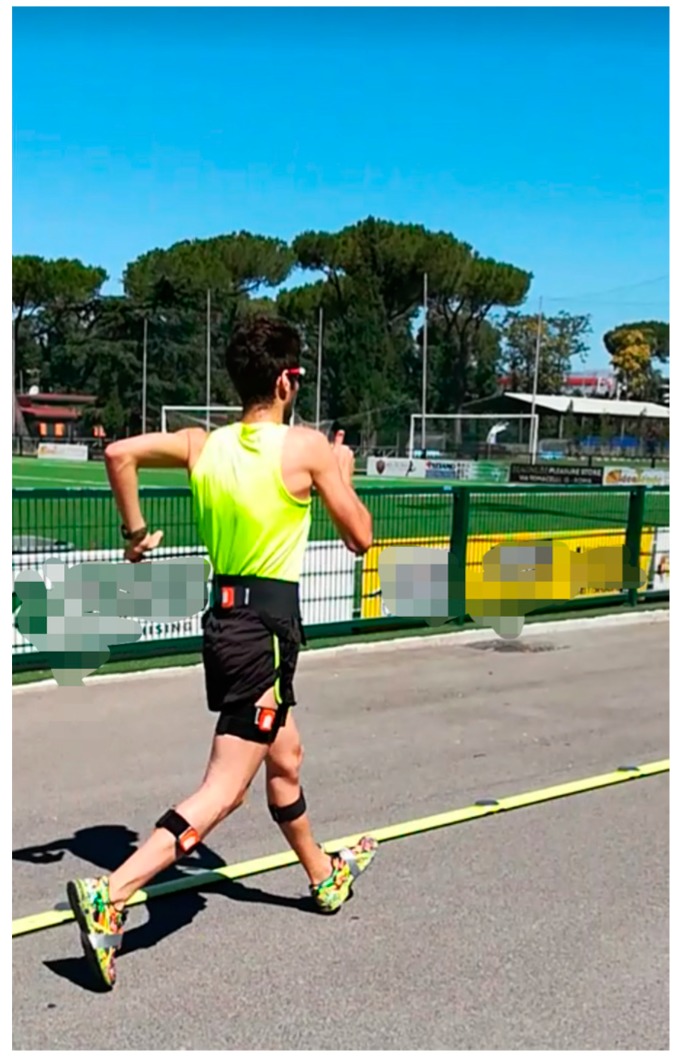
Placement of IMUs (orange probes) on an athlete during the experimental procedure.

IMUs were placed on the hip, left and right thigh, left and right shank, and left and right foot, as shown in Figure 5. Sensor alignment was performed manually by the same expert operator and each IMU was fixed with elastic straps to limit relative movements between sensor and body segment.

Athletes were asked to perform one task in an ad hoc path of 250 m, 100 m of straight line, and two curves with a radius about 4 m, which is faithful to the Olympic paths [22]. The experimental task consisted of three laps of the path. Specifically, subjects were asked to race walk in three different conditions: (i) at preferred race pace during the first lap (legal race); (ii) simulating the LC fault during the second lap (illegal race); and (iii) simulating the KB fault during the third lap (illegal race). The experimental task was repeated two times per subject. The operator notified the athlete at the start of each lap on the race walking condition to be performed. During both repetitions, linear acceleration and angular velocity of the lower-limb segments in the three anatomical planes (sagittal, frontal, and transversal) were acquired at a sampling rate of 60 Hz. 

A further IMU was assigned to the coach, acting as a referee. The coach was asked to rotate the sensor 180° in correspondence with the actual transition among race walking conditions. In fact, the transition among race walking conditions did not occur instantaneously when the operator announced the end of each lap. Even though the walkers were asked to perform the entire lap in the same condition, the coach was asked to indicate any transition among race walking conditions even within the same lap. Any stride related to a specific race walking condition not required during that lap was discarded during the data analysis. Vertical acceleration of the IMU assigned to the coach was acquired during both repetitions. 

### 3.3. Data Analysis

Considering all the IMUs, a total of 42 kinematic variables were acquired: linear acceleration and angular velocity related to each anatomical plane (6) for each sensor (7). Acquired data were processed with MATLAB (MathWorks, 2012b, Natick, MA, USA). Both linear acceleration and angular velocity were treated with a fourth-order low-pass Butterworth filter with the cut-off frequency set at 20 Hz. Data gathered from the IMU assigned to the coach were used to identify the transitions among the three race walking conditions by considering the sign variation related to vertical acceleration due to the IMU’s rotation of 180°. The identified transitions were used to create the reference sequence (S_ref_) of walking race conditions in order to label each kinematic variable with the three race walking conditions. Each signal related to each walking condition was further partitioned into strides following the algorithm proposed by Salarian et al. [39]. Each stride was defined as the time interval between two consecutive heel strikes of the same foot. The first and the last three strides of each race walking condition were eliminated to guarantee the perfect adjustment of the athlete to the new race walking condition; then, the same number of strides for the three race walking conditions was selected. Finally, each stride defined according to the Salarian algorithm was resampled to 100 samples. 

Successively, seven features were extracted from the kinematic variables for each stride, as reported in Table 2.

A matrix *n* × *f* was obtained for each variable, where *n* is the number of strides considering all three conditions of race walking, and *f* is the number of features. For each body segment, three datasets of features were constructed by considering: (i) only the linear accelerations, (ii) only the angular velocities, and (iii) the linear accelerations and angular velocities together. Considering the symmetry of race walking, the data gathered from the right and left thighs, shanks, and feet were considered together. Thus, a total of 12 datasets of features were gathered: 4 body segments (pelvis, thighs, shanks, and feet) × 3 combinations of signals (linear accelerations, angular velocities, and the two variables together) for each repetition and each athlete. 

Toward the aim of the study, we comparatively examined nine classifiers, which were, as reported in Section 3.1, DT_f_, SVM_l_, SVM_q_, SVM_c_, kNN_f_, kNN_c_, kNN_cu_, kNN_w_, and ANN. Their performance was evaluated by means of a cross-validation using the first repetition as training and the second one as test, and vice versa [40]. In total, we tested 108 machine-learning algorithms for each athlete, as combinations of: 4 body segments × 3 datasets × 9 classifiers. The following nomenclature was chosen for indicating the 108 machine-learning algorithms:

(5) Cds

where:

- C is the name of the classifier, which includes DT_f_, SVM_l_, SVM_q_, SVM_c_, kNN_f_, kNN_c_, kNN_cu_, kNN_w_, and ANN;
- s is the segment, which is pelvis (PL), thigh (TH), shank (SH), and foot (FT); and
- d is the dataset, which is composed of signals of accelerations (a), angular velocities (ω), and accelerations and angular velocities together (aω).

### 3.4. Performance Evaluation

The performance of each machine-learning algorithm was computed by comparing the estimated race walking condition sequence with the S_ref_ and, then, by averaging the results of the cross-validation procedure. Finally, a 3 × 3 confusion matrix was obtained for each subject.

**Figure 6:**
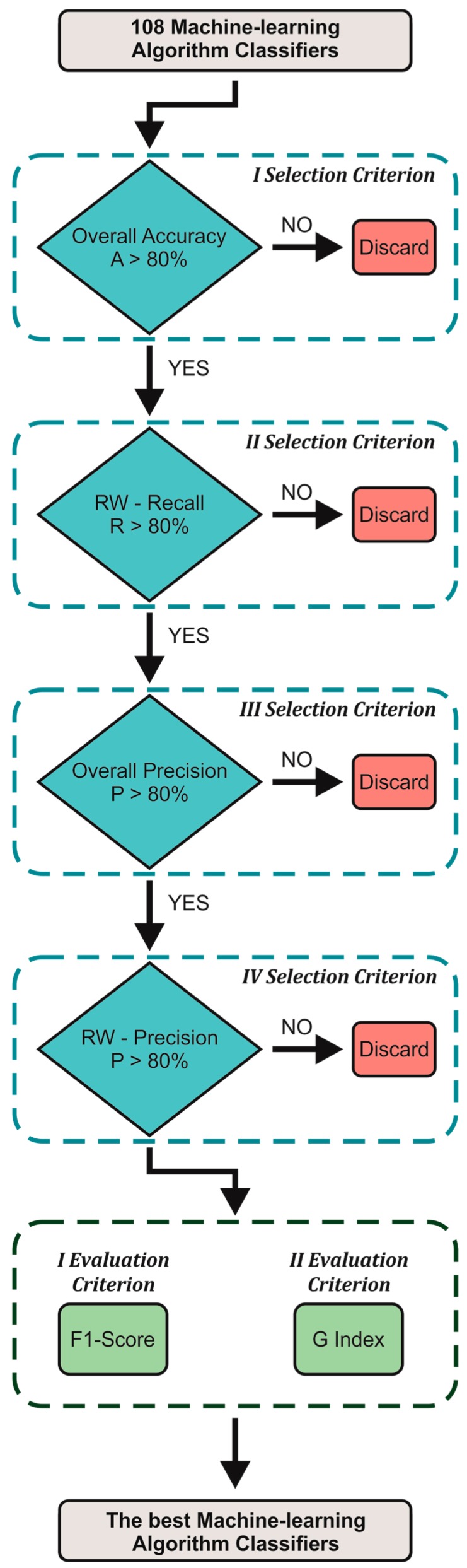
Flow chart for the identification of the best-performing classifiers. RW stands for race walking condition.

In order to individuate the best-performing machine-learning algorithms among the 108 tested, we applied four selection criteria and two evaluation criteria based on the computation of synthetic indices from the analysis of the confusion matrix (Figure 6). More specifically, the selection criteria were based on specific performance thresholds to reach in order to make an initial skimming, while the evaluation criteria allowed for selecting the best-performing classifiers. 

*First Selection Criterion—Overall Accuracy*

The overall accuracy (A) is the ratio of the correctly predicted race walking strides to the total race walking strides, and it is an overall index that considers all three classes together. It was computed by Equation (6):

(6) $$A=\frac{\mathrm{TP}+\mathrm{TN}}{\mathrm{TP}+\mathrm{FP}+\mathrm{TN}+\mathrm{FN}}$$

where TP, TN, FP, and FN are the true positive, true negative, false positive, and false negative, respectively. The overall accuracy was computed individually for each athlete and classifier. Then, the mean and standard deviation were obtained for each classifier by averaging the accuracy values across athletes. The first selection step was passed only by the classifiers that reached an overall accuracy of at least 0.80. This threshold was chosen since it is a typical value for considering a classifier as good or optimum [41,42] and it is also the accuracy reached by previous works published on the automatic detection of illegal steps in race walking [22,25]. 

*Second Selection Criterion—Recall*

The recall (R) is the ratio of TP to the sum of TP and FN, and it is an index typical for each class of the classification. It was computed by Equation (7):

(7) $$R=\frac{\mathrm{TP}}{\mathrm{TP}+\mathrm{FN}}\;.$$

The recall was computed individually for each athlete, each race walking condition, and each classifier that passed the first selection step. Then, the mean and standard deviation for each race walking condition and classifier were gathered by averaging the recall values across the athletes. The mean value of R across the three walking conditions coincided with the overall accuracy.

The second selection criterion was passed only by the classifiers that reached a recall value of at least 0.80 for all the three race walking conditions. This threshold guaranteed a good or optimum classification related to, not only the overall model, but also each race walking condition [41,42].

By the two reported selection criteria, only the robustness of the classifier to type II errors [43] was considered. However, robustness to type I errors (i.e., avoiding the false positive) represents an essential requirement for the design of a classifier for this application. Thus, criteria on precision value had to be considered. 

*Third and Fourth Selection Criteria—Precision*

The positive predicted value or precision (P) is the ratio of TP to the sum of TP and FP, and it is an index typical for each class of the classification. It was computed by Equation (8):

(8) $$P=\frac{\mathrm{TP}}{\mathrm{TP}+\mathrm{FP}}\;.$$

The precision was computed individually for each athlete, each race walking condition, and each classifier that passed the second selection criterion. Then, the mean and standard deviation for each race walking condition and classifier were gathered by averaging the precision values across the athletes. In addition, the mean and standard deviation across the three race walking conditions were computed to determine the overall precision. 

The third selection step was passed only by the classifiers that reached an overall precision value of at least 0.80. Then, the fourth selection criterion consisted of a precision value of at least 0.80 for each race walking condition. By taking into account theese two selection criteria, robustness to type I errors [43] was also evaluated. 

*First Evaluation Criterion—F1-Score*

The F1-score was computed individually for each athlete, each race walking condition, and each classifier that passed the previous four selection criteria. The F1-score is the harmonic average of R and P, and it is an index typical for each class of the classification. It was computed by Equation (9):

(9) $$F1\text{-}\mathrm{score}=2\cdot \frac{\left(R\cdot P\right)}{\left(R+P\right)}.$$

The mean and standard deviation for each race walking condition and classifier were gathered by averaging the F1-score values across the athletes. In addition, the mean and standard deviation across the three race walking conditions were computed to determine the overall F1-score. A one-way repeated measurements ANOVA test was performed to find noteworthy differences among the remaining classifiers, individually for the F1-score related to each race walking condition and the overall F1-score. Statistical difference was set at 0.05. When significant differences were found, a Bonferroni’s test for multiple comparisons was performed. All data were first tested for normality with the Shapiro–Wilk test. The potential presence of statistical differences permitted the identification of the best-performing classifier in the identification of both faults during race walking. 

*Second Evaluation Criterion—Goodness Index*

For the classifiers that passed the four selection criteria, a simplified 2 × 2 confusion matrix was constructed considering all the athletes together. In particular, it was obtained by considering the two faults together, evaluating if the performance increased when it was required to discriminate regular race walking from illegal steps, regardless of the type of fault. The performance of the obtained simplified classifiers was assessed by the overall accuracy and the goodness index (G). G was computed by Equation (10):

(10) $$G=\sqrt{{\left(1-\mathrm{TP}\right)}^{2}+{\left(1-\mathrm{TN}\right)}^{2}}.$$

G represents the Euclidean distance between the evaluated point in the ROC (Receiver operating characteristic) space and the point [0 1], which represents the perfect classifier. G can assume values between 0 and $\sqrt{2}$, and a classifier can be considered as: (i) optimum when G ≤ 0.25; (ii) good when 0.25 < G ≤ 0.70; (iii) random if G = 0.70; and (iv) bad if G > 0.70 [42,44]. By analyzing G value results, the best-performing classifier in the identification of faults, regardless of their type, could be evaluated. 

## 4. Results and Discussions

A total of 972 strides per race walking condition were examined considering all of the athletes. The average value per athlete was 121 ± 23 strides for each condition. 

*First Selection Criterion—Overall Accuracy*

The mean and standard deviation of the overall accuracy are reported in Table 3.

Thirty out of the 108 classifiers passed the first selection criterion. More specifically, 15 classifiers were fed with the acceleration and the remaining 15 were based on the combination of linear accelerations and angular velocities. Most of the classifiers that passed the first selection were implemented with data related to the IMUs placed on shanks and feet. 

No classifier only fed with the angular velocity reached an overall accuracy equal to or greater than the set threshold. Thus, we observed that features based on angular velocity did not present significant differences among the three race walking conditions. Consequently, the automatic recognition of faults based only on angular velocity data is unfeasible. Specific literature, in fact, has demonstrated that linear acceleration is the most appropriate variable to consider when seeking the recognition of physical human activities [30]. Conversely, angular velocity reveals itself as the most suitable variable for detecting gait events for the classification of gait phases, as reported in [16,45,46]. Thus, this suggests using angular velocity to detect gait events in order to evaluate the patterns related to subphases of the stride and linear accelerations to automatically detect faults. Furthermore, we observed that inserting features related to the angular velocity into the classifier fed with linear acceleration did not always improve performance. This confirms that using a higher data dimensionality in a classification process does not automatically benefit the performance and robustness of the classification method [16].

Regarding the specific machine-learning algorithms, SVMqaSH reached the highest overall accuracy value (0.90). Moreover, the SVMs and k-means appeared to be the most stable across subjects due to the low values of standard deviation. These outcomes suggest that k-means and support vector machines reached similar or greater performance, confirming them as the best-performing classifiers among the supervised machine-learning algorithms in the automatic detection of physical activities [47].

Conversely, no algorithm based on a decision tree passed the accuracy threshold. This might be ascribed to the classification logic based on the identification of thresholds. As previously demonstrated [9], threshold-based classifiers present lower robustness among subjects. Moreover, machine-learning algorithms, such as kNN, SVM, and ANN, are more effective at discriminating among activities with respect to the DT. Thus, they should be preferred when the final classification is the only output of interest [48]. Conversely, the decision tree represents an optimum trade-off in applications requiring real-time modifications of model parameters, considering that DT is the only “white box” classifier among those tested, which also implies easier manipulation of the model parameters after model initialization [48]. 

*Second Selection Criterion—Recall*

The recall mean and standard deviation values of the classifiers that passed the first selection for each race walking condition are reported in Table 4. Fourteen out of the 30 classifiers passed the selection based on the recall value. The highest value of recall was reached by the SVM fed with linear acceleration data of shanks in the classification of regular race walking (0.91). No tested kNN_f_ passed the selection.

Regarding the pelvis and thighs, no classifier passed the second selection; more specifically, they showed a recall value under the set threshold in the classification of the KB fault. In addition, a higher variability was found for the classifiers based on the data related to pelvis and thighs, suggesting that good performance cannot be reached with data gathered from all athletes.

Although the pelvis acceleration represents the variable generally considered for the discrimination of loss of contact in other studies [7,23,25], based on our results, it had weak performance in the recognition of the knee-bent fault. We ascribe this finding to a different approach. In the cited studies, only the loss of contact detection was performed; thus, the linear acceleration related to both the pelvis and thigh can be considered useful for the identification of the flight time during race walking but not when seeking to automatically detect the knee-bent fault as well. 

Focusing on the shank district, six classifiers passed the selection. The remaining three (i.e., kNNfaSH, kNNwaSH, and SVMlaωSH) showed a recall value under the set threshold in the classification of regular race walking. Concerning feet, eight classifiers passed the selection.kNNfaFT, kNNwaFT, and kNNwaωFT were discarded for low recall values in the classification of the LC fault, while kNNcoaωFT and ANNaωFT were discarded for low recall values in the classification of the KB fault.

These findings might be ascribed to the greater variability among the three race walking conditions. In fact, the linear acceleration of feet and shank, unlike the others, presented different signal patterns among normal race walking, loss of contact, and knee-bent. Generally, the classifiers fed with the combination of linear acceleration and angular velocity showed greater variability in the classification of regular walking race (standard deviation ≥ 0.10).

*Third and Fourth Selection Criteria—Precision*

The means and standard deviations of the precision of the overall classification model and each race walking condition are reported in Table 5. 

All remaining classifiers passed the third selection criterion. The highest value of overall precision (0.90) was reached by SVMqaSH and SVMcaSH. Generally, a lower inter-subject variability of the precision was observed with respect to the accuracy, as demonstrated by the values of standard deviation always being lower than 0.10.

Regarding the fourth criterion, seven classifiers, which were all based on the SVM classifier, passed the selected threshold. The highest value of precision (0.92) was reached by SVMqaSH and SVMcaSH for the classification of the LC fault. The remaining classifiers were four for the shanks and three for the feet. Six out of the seven deleted classifiers did not achieve sufficient precision in the classification of regular race walking, while the remaining one did not in the classification of the loss-of-contact fault. 

No tested kNN or ANN reached a precision value equal to or greater than 0.80 for all three race walking conditions. Thus, these classifiers were not robust with respect to type I errors, especially when the focus was on the discrimination of normal race walking. The lack of robustness led to a large number of false positives, which occurred when the classifier estimated that the athlete was correctly race walking when it was not so. As the actual aim of the study was fault detection, we can affirm that kNN and ANN, although having good results in terms of accuracy, should be avoided due to the low precision. 

Conversely, classifiers based on the SVM approach should be implemented when the discrimination of faults during walking has to be performed with high accuracy and high precision. This finding points to the SVM as the most appropriate classifier to use, confirming the high performance generally found for this classifier in the identification of physical human activities [33]. 

The obtained performance values were in line with those reported in other studies on race walking for the identification of loss of contact [7,8]. Furthermore, a similar performance was also obtained here for the discrimination of knee-bent faults, which has never been evaluated in the literature via machine-learning approach.

*First Evaluation Criterion—F1-Score*

The mean and standard deviation values of the F1-score for the overall classification in each race walking condition are reported in Table 6.

No statistical differences were found for the overall F1-score and the F1-score computed in the classification of regular race walking and LC faults. Regarding the classification of KB faults, the F1-score related to SVMqaSH was statistically greater than those of all the remaining classifiers, with the exception of SVMcaSH. 

**Figure 7:**
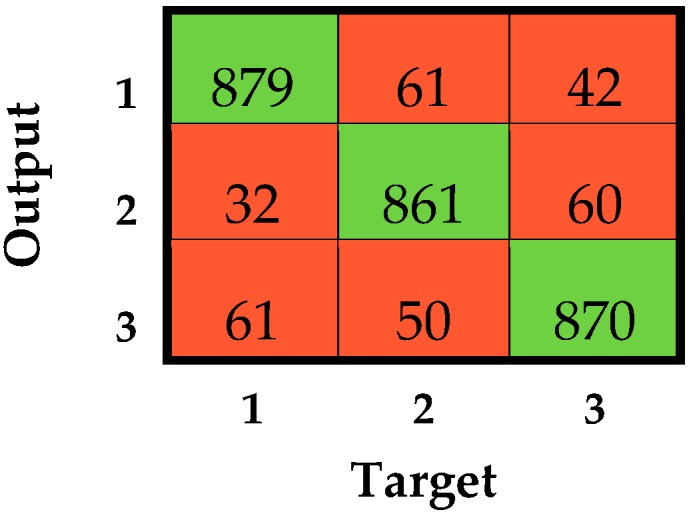
Confusion matrix related to the SVMqaSH considering all subjects together. 1, 2, and 3 stand for regular, loss of contact, and knee-bent condition, respectively. Numbers in green cells represent the correct classifications, while those in red cells are misclassifications.

In terms of overall F1-score, as no statistical difference was found, the seven machine-learning classifiers that passed all previous thresholds on accuracy and precision can be considered equivalent in the identification of faults during race walking. However, some considerations can be reported in order to identify the best-performing classifiers. Firstly, the classifiers fed with the linear accelerations related to the feet showed the highest value of standard deviation relative to the F1-score for the loss of contact, suggesting that low performance was obtained in some athletes. These classifiers presented lower robustness among subjects and, thus, their use is not advisable. In fact, the implementation of these classifiers during a race walking competition could imply a greater misclassification rate for some athletes, leading to an unfair judgment. Secondly, the addition of features related to the angular velocity could be avoided, since no significant improvements were obtained. In fact, the addition of data causes a greater computational load that can affect the real-time identification of faults during an official competition. From this perspective, the quadratic and cubic SVMs fed with the linear acceleration of shanks showed the best values in all of the computed metrics. However, it is known that the prediction speed, memory usage, and interpretability of the results worsen by increasing the order of the kernel function [49]. Consequently, the SVMqaSH could be assumed to be the best-performing classifier for achieving the aim of the study. The confusion matrix related to the SVMqaSH considering all subjects together is reported in Figure 7. 

*Second Evaluation Criterion—Goodness Index*

The overall accuracy and the goodness index values related to the simplified classifiers are reported in Table 7. 

**Figure 8:**
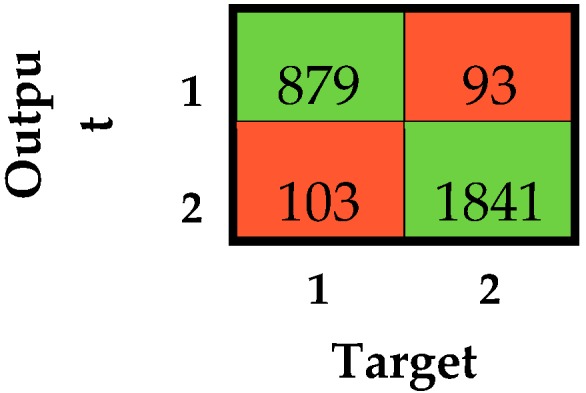
Confusion matrix related to the simplified SVMqaSH considering all subjects together. 1 and 2 stand for regular and irregular race walking, respectively. Numbers in green cells represent the correct classifications, while those in red cells are misclassifications.

The simplified version of the classifiers allowed for increasing the obtained performance, with an overall accuracy up to 0.93 that was always equal to or greater than 0.90. All the classifiers showed a G value within the range of optimum classifiers. This finding suggests that the majority of incorrect classifications were due to a misclassification between loss of contact and knee-bent. However, a misclassification between loss of contact and knee-bent does not influence the regularity of the race walking competition since a warning is given to an athlete regardless of the nature of the fault. This consideration strengthens the preference of the SVMqaSH as the best-performing classifier. The confusion matrix related to the simplified model SVMqaSH considering all subjects together is reported in Figure 8. 

The higher performance obtained with the simplified version could offer a starting point to further study the development of a different sensor system to separate the detection of LC from KB faults. For instance, the addition of a pressure insole could allow for the evaluation of LC faults, while only KB faults would be detected through the machine-learning algorithm.

Such an approach could also be adapted to other sports in which the judgment of specific movements is still performed by the human eye, such as artistic gymnastics, fencing, boxing, and wrestling, or to monitor specific techniques in order to prevent injuries and/or evaluate an athlete’s performance evolution. In addition, the proposed methodology can be a useful tool to embed in smart mobile systems for applications in clinics; in fact, the feasibility of a machine-learning approach to investigate anomalies in human gaits has already been demonstrated [41,50].

## 5. Conclusions

In this paper, we investigated the feasibility of using machine-learning algorithms fed with inertial data related to lower-limb segments in the automatic identification of race walking faults. In particular, a comparison among the performance of 108 classifiers was conducted. The outcomes of our study endorse the quadratic support vector machine fed with the linear acceleration related to the shanks as the best-performing classifier for the identification of both LC and KB faults. The results raise the possibility of developing a wearable sensor that could be a useful tool for helping the judgement of race walking regularity during training and/or competition. 

Future works will include online testing of the methodology, the feasibility of avoiding subject-specific training of the classifier used in this paper, as well as the design and development of a wearable smart device that can be used during race walking competitions.

## 6. Patents

A National Patent resulting from the work reported in this manuscript is pending. (Cappa P., Palermo E., Rossi S. and Taborri J. “Procedimento e dispositivo per rilevare condizioni di marcia durante la marcia di un atleta” 8 June 2017).

## Figures and Tables

**Table 1:** Specifics of used the inertial measurement units (IMUs).

	Gyroscope	Accelerometer
**Full Scale**	±1200 °/s	±160 m/s^2^
**Linearity**	0.1% FS	0.2% FS
**Stability**	20 °/h	-
**Noise**	0.05 °/s/√Hz	0.003 m/s^2^/√Hz
**Misalignment error**	0.1°	0.1°
**Bandwidth**	100 Hz	100 Hz
**Battery autonomy**	8 h	

**Table 2:** List of features extracted from each stride of each acquired variable.

Time Features	Frequency Features
Mean Standard deviation Maximum Minimum	Height of main peak of the autocorrelation Height of the second peak of the autocorrelation Position of the second peak of the autocorrelation

**Table 3:** Mean and standard deviation of the overall accuracy. The green cells represent classifiers that passed the first selection; others are highlighted in red.

Variable	Segment	DT_f_	SVM_l_	SVM_q_	SVM_c_	kNN_f_	kNN_c_	kNN_cu_	kNN_w_	ANN
Acceleration	Pelvis	0.70 (0.18)	0.79 (0.20)	0.76 (0.21)	0.75 (0.21)	0.73 (0.21)	0.73 (0.21)	0.73 (0.18)	0.73 (0.23)	0.75 (0.18)
	Thighs	0.75 (0.05)	0.83 (0.11)	0.83 (0.10)	0.83 (0.10)	0.77 (0.09)	0.77 (0.11)	0.76 (0.09)	0.78 (0.10)	0.79 (0.10)
	Shanks	0.77 (0.07)	0.84 (0.06)	0.90 (0.02)	0.88 (0.03)	0.80 (0.08)	0.79 (0.10)	0.79 (0.07)	0.81 (0.03)	0.79 (0.08)
	Feet	0.74 (0.06)	0.86 (0.05)	0.87 (0.05)	0.87 (0.03)	0.83 (0.04)	0.82 (0.05)	0.79 (0.04)	0.82 (0.03)	0.86 (0.04)
Angular Velocity	Pelvis	0.66 (0.16)	0.72 (0.19)	0.76 (0.21)	0.75 (0.21)	0.73 (0.22)	0.73 (0.21)	0.72 (0.20)	0.74 (0.22)	0.69 (0.21)
	Thighs	0.70 (0.10)	0.79 (0.09)	0.79 (0.09)	0.79 (0.08)	0.75 (0.09)	0.76 (0.09)	0.73 (0.08)	0.76 (0.10)	0.72 (0.13)
	Shanks	0.69 (0.06)	0.73 (0.12)	0.74 (0.12)	0.74 (0.12)	0.73 (0.08)	0.73 (0.10)	0.70 (0.09)	0.74 (0.08)	0.71 (0.08)
	Feet	0.71 (0.06)	0.76 (0.15)	0.75 (0.15)	0.74 (0.18)	0.73 (0.17)	0.74 (0.18)	0.70 (0.16)	0.74 (0.17)	0.73 (0.15)
Acceleration and Angular Velocity	Pelvis	0.72 (0.16)	0.80 (0.18)	0.81 (0.18)	0.79 (0.20)	0.78 (0.19)	0.77 (0.20)	0.75 (0.19)	0.78 (0.19)	0.74 (0.22)
	Thighs	0.76 (0.08)	0.81 (0.10)	0.82 (0.11)	0.80 (0.10)	0.79 (0.08)	0.79 (0.10)	0.74 (0.09)	0.79 (0.08)	0.76 (0.11)
	Shanks	0.78 (0.08)	0.80 (0.10)	0.88 (0.04)	0.87 (0.04)	0.79 (0.07)	0.79 (0.08)	0.78 (0.07)	0.81 (0.07)	0.78 (0.10)
	Feet	0.71 (0.09)	0.85 (0.08)	0.83 (0.15)	0.83 (0.14)	0.79 (0.14)	0.80 (0.13)	0.75 (0.18)	0.81 (0.11)	0.80 (0.14)

**Table 4:** Mean and standard deviation values of recall for the classifiers that passed the first selection criterion. The green cells represent classifiers that passed the second selection criterion; the others are highlighted in red. “-“ indicates a classifier that did not pass previous criteria.

	Condition	SVMla	SVMqa	SVMcaω	kNNfa	kNNcoa	kNNwa	ANNa	SVMlaω	SVMqaω	SVMcaω	kNNcoaω	kNNwaω	ANNaω
Pelvis	Regular	-	-	-	-	-	-	-	0.81 (0.31)	0.78 (0.30)	-	-	-	-
	LC	-	-	-	-	-	-	-	0.82 (0.16)	0.84 (0.16)	-	-	-	-
	KB	-	-	-	-	-	-	-	0.78 (0.07)	0.80 (0.18)	-	-	-	-
Thigh	Regular	0.87 (0.11)	0.86 (0.11)	0.87 (0.12)	-	-	-	-	0.79 (0.24)	0.81 (0.20)	0.83 (0.17)	-	-	-
	LC	0.90 (0.14)	0.90 (0.04)	0.89 (0.04)	-	-	-	-	0.91 (0.07)	0.90 (0.06)	0.83 (0.16)	-	-	-
	KB	0.71 (0.29)	0.72 (0.29)	0.73 (0.27)	-	-	-	-	0.74 (0.30)	0.75 (0.30)	0.74 (0.29)	-	-	-
Shank	Regular	0.80 (0.14)	0.91 (0.05)	0.90 (0.05)	0.79 (0.23)	-	0.77 (0.25)	-	0.71 (0.28)	0.86 (0.10)	0.86 (0.10)	-	0.81 (0.18)	-
	LC	0.85 (0.12)	0.89 (0.06)	0.88 (0.06)	0.81 (0.08)	-	0.82 (0.06)	-	0.85 (0.10)	0.90 (0.05)	0.89 (0.05)	-	0.81 (0.09)	-
	KB	0.88 (0.05)	0.90 (0.04)	0.88 (0.05)	0.81 (0.14)	-	0.83 (0.11)	-	0.83 (0.07)	0.86 (0.06)	0.86 (0.05)	-	0.81 (0.09)	-
Foot	Regular	0.86 (0.15)	0.87 (0.14)	0.89 (0.08)	0.90 (0.13)	0.85 (0.19)	0.89 (0.14)	0.85 (0.15)	0.81 (0.20)	0.80 (0.28)	0.81 (0.26)	0.82 (0.17)	0.85 (0.17)	0.82 (0.17)
	LC	0.85 (0.05)	0.87 (0.07)	0.87 (0.08)	0.75 (0.14)	0.83 (0.10)	0.76 (0.14)	0.85 (0.07)	0.84 (0.07)	0.87 (0.09)	0.84 (0.08)	0.82 (0.08)	0.76 (0.10)	0.83 (0.09)
	KB	0.88 (0.06)	0.86 (0.09)	0.85 (0.08)	0.81 (0.12)	0.80 (0.14)	0.82 (0.10)	0.88 (0.06)	0.89 (0.06)	0.81 (0.22)	0.84 (0.16)	0.76 (0.22)	0.82 (0.17)	0.76 (0.29)

**Table 5:** Mean and standard deviation of the overall precision and the precision for each condition. The green cells represent classifiers that passed the third and fourth selection criteria; the others are highlighted in red.

		Third Criterion	Fourth Criterion		
Segment	Classifier	Overall Precision	Regular	Loss of Contact	Knee-Bent
Shanks	SVMlaSH	0.85 (0.08)	0.79 (0.17)	0.93 (0.05)	0.82 (0.10)
	SVMqaSH	0.90 (0.01)	0.89 (0.05)	0.92 (0.05)	0.89 (0.04)
	SVMcaSH	0.90 (0.02)	0.89 (0.04)	0.92 (0.05)	0.89 (0.04)
	SVMqaωSH	0.89 (0.02)	0.88 (0.04)	0.91 (0.06)	0.90 (0.05)
	SVMcaωSH	0.89 (0.02)	0.87 (0.04)	0.91 (0.06)	0.89 (0.05)
	kNNwaωSH	0.85 (0.08)	0.79 (0.05)	0.94 (0.05)	0.82 (0.14)
Feet	SVMlaFT	0.88 (0.06)	0.84 (0.06)	0.95 (0.04)	0.85 (0.10)
	SVMqaFT	0.89 (0.05)	0.86 (0.07)	0.94 (0.07)	0.87 (0.08)
	SVMcaFT	0.89 (0.03)	0.86 (0.07)	0.93 (0.07)	0.88 (0.07)
	kNNcoaFT	0.85 (0.05)	0.79 (0.05)	0.89 (0.06)	0.85 (0.07)
	ANNaFT	0.83 (0.05)	0.82 (0.06)	0.79 (0.09)	0.88 (0.05)
	SVMlaωFT	0.86 (0.07)	0.79 (0.06)	0.93 (0.07)	0.84 (0.12)
	SVMqaωFT	0.83 (0.04)	0.78 (0.14)	0.88 (0.19)	0.82 (0.17)
	SVMcaωFT	0.84 (0.05)	0.79 (0.10)	0.89 (0.18)	0.84 (0.12)

**Table 6:** Mean and standard deviation of the F1-score for both the overall classification and each race walking condition. Superscripts indicate statistical differences among classifiers.

Segment	Classifier		Race Walking Condition		
		Overall	Regular	Loss of Contact	Knee-Bent
Shanks	(1) SVMqaSH	0.89 (0.03)	0.89 (0.05)	0.90 (0.05)	0.89 ^3,4,5,6,7^ (0.04)
	(2) SVMcaSH	0.89 (0.03)	0.89 (0.04)	0.90 (0.05)	0.88 ^6,7^ (0.03)
	(3) SVMqaωSH	0.88 (0.03)	0.87 (0.06)	0.90 (0.04)	0.86 ^1^ (0.05)
	(4) SVMcaωSH	0.88 (0.03)	0.87 (0.06)	0.90 (0.04)	0.87 ^1^ (0.05)
Feet	(5) SVMlaFT	0.85 (0.06)	0.82 (0.14)	0.89 (0.05)	0.85 ^1^ (0.07)
	(6) SVMqaFT	0.86 (0.05)	0.83 (0.12)	0.90 (0.06)	0.85 ^1,2^ (0.05)
	(7) SVMcaωFT	0.87 (0.03)	0.86 (0.06)	0.89 (0.06)	0.85 ^1,2^ (0.04)

**Table 7:** Overall accuracy and goodness index related to the suitable classifiers that passed all previous selection criteria.

Segment	Classifier	Overall Accuracy	Goodness Index
Shanks	SVMqaSH	0.93	0.11
	SVMcaSH	0.92	0.12
	SVMqaωSH	0.91	0.15
	SVMcaωSH	0.91	0.15
Feet	SVMlaFT	0.90	0.15
	SVMqaFT	0.90	0.15
	SVMcaωFT	0.90	0.13
